# Lifestyle and Breast Cancer: Prevention and Treatment Support

**DOI:** 10.3390/cancers17172830

**Published:** 2025-08-29

**Authors:** Alicja Ewa Ratajczak-Pawłowska, Karolina Jezierska, Aleksandra Szymczak-Tomczak, Agnieszka Zawada, Anna Maria Rychter, Kinga Skoracka, Agnieszka Dobrowolska, Iwona Krela-Kaźmierczak

**Affiliations:** 1 Laboratory of Nutrigenetics, Department of Gastroenterology, Dietetics and Internal Diseases, Poznan University of Medical Sciences, 61-701 Poznan, Poland; arychter@ump.edu.pl (A.M.R.); ikrela-kazmierczak@ump.edu.pl (I.K.-K.); 2 Department of Gastroenterology, Dietetics and Internal Diseases, Poznan University of Medical Sciences, 61-701 Poznan, Poland; aszymczaktomczak@ump.edu.pl (A.S.-T.); a.zawada@ump.edu.pl (A.Z.); kingskoracka@gmail.com (K.S.); agdob@ump.edu.pl (A.D.); 3 Moles Diagnostic Center, 60-821 Poznan, Poland; karolina.jezierska95@gmail.com; 4 Doctoral School, Poznan University of Medical Sciences, 61-701 Poznan, Poland

**Keywords:** breast cancer, prevention, nutrition, gastrointestinal microbiome, lifestyle

## Abstract

Breast cancer is the most common malignant tumor among women. It is a significant health and social issue affecting 2.3 million women worldwide. Breast cancer is caused by various factors, including gender, age, race, genetics, hormonal balance, obesity, alcohol and many others. A crucial issue related to breast cancer is the impact of diet on the development of the disease. Dietary fats play a key role. Saturated fatty acids and trans fatty acids increase the risk of breast cancer, while polyunsaturated fatty acids have a protective effect. A high-protein diet reduces the risk of breast cancer and improves prognosis among patients. The role of carbohydrates remains unclear, but women may benefit from reducing their intake of high glycemic index foods. Among the macronutrients influencing the development and progression of breast cancer are calcium and magnesium. Adopting a Mediterranean diet may offer benefits. Among the factors influencing the development of breast cancer, the gastrointestinal microbiota is also noteworthy. Regular physical activity can reduce the risk of developing breast cancer, support treatment, reduce side effects and improve patients’ quality of life. Chronic stress also contributes to the development of breast cancer by affecting the nervous, hormonal and immune systems, disrupting the body’s homeostasis.

## Simple Summary

There are known genetic factors associated with breast cancer. However, there are also many lifestyle factors which may affect the risk of breast cancer. The modification of diet, physical activity and stress management may decrease the risk of disease and improve the prognosis in women with breast cancer.

## 1. Introduction

### 1.1. Breast Cancer

Breast cancer is the most common malignant tumor diagnosed in women. This type of cancer typically originates in the lactiferous ducts or the lobules responsible for milk production within the breast. According to data from the World Health Organization (WHO), in 2022 [1], breast cancer was diagnosed in 2.3 million women worldwide, resulting in 670,000 deaths. Although predominantly a female disease, it also affects approximately 0.5–1% of men [1]. In Poland, data from the National Cancer Registry for 2020 indicate that breast cancer accounted for 24% of all cancer cases and 15% of all cancer-related deaths among women [2]. The risk of developing breast cancer increases with age. Approximately 80% of patients are over the age of 50, while 40% are aged 65 or older [3,4]. Risk factors for breast cancer can be divided into non-modifiable and modifiable categories. Non-modifiable factors include female sex, older age, a positive family history of breast cancer and the presence of *BRCA1* or *BRCA2* mutations. Modifiable risk factors include obesity, physical inactivity, alcohol consumption, hormone replacement therapy and several others [4].

Breast cancer encompasses a heterogeneous group of tumors that vary in morphology, symptoms and clinical course. Histologically, breast cancers are classified into non-invasive (in situ) and invasive types. The World Health Organization recognizes at least 18 histological subtypes of breast cancer, with the most common being invasive breast cancer of no special type (NST), which accounts for approximately 80% of cases. A molecular classification is also used, distinguishing luminal breast cancer (types A and B), HER2-enriched breast cancer, triple-negative breast cancer and claudin-low breast cancer [5]. Among these, triple-negative breast cancer has the worst prognosis due to its lack of estrogen, progesterone and HER2 receptor expression, as well as its aggressive clinical behavior [6]. An important aspect of breast cancer control is the use of screening programs aimed at detecting the disease at an early stage. In a systematic review by Ren W. et al. (2022) [7], 23 international guidelines published between 2010 and 2021 across 11 countries or regions were analyzed to compare recommendations and screening methods. Most guidelines advocated mammographic screening every 1–2 years for women aged 40–74 at average risk and annual mammography or magnetic resonance imaging (MRI) for high-risk populations, starting at a younger age.

The review was based primarily on guidelines from high-income countries in the United States and Europe, reflecting a lack of available data on screening in low- and middle-income countries. This gap likely stems from insufficient national evidence on breast cancer screening and a lack of resources to develop such guidelines [7]. In Poland, as of 1 November 2023, a Breast Cancer Prevention Program is available, funded by the National Health Fund (NFZ). Under this initiative, women aged 45 to 74 are eligible for free mammographic screening every 2 years [8].

Screening programs play a crucial role in the diagnosis and treatment of breast cancer. Participation by at least 70% of the eligible population has been shown to reduce breast cancer mortality by 40% [9]. Survival rates for breast cancer vary significantly by country. In high-income countries, the five-year survival rate generally exceeds 90%. In contrast, in sub-Saharan Africa, it averages 66%, with rates as low as 12% in Uganda for cases diagnosed between 2008 and 2015 [10]. These disparities highlight the importance of widespread preventive programs and equitable access to medical care in combating this disease. Breast cancer remains a major global health and social concern, affecting millions of women worldwide. The WHO Global Breast Cancer Initiative seeks to lower worldwide breast cancer mortality by 2.5% each year, potentially saving 2.5 million lives between 2022 and 2040. Achieving this goal relies on health promotion, timely diagnosis and comprehensive approaches to treatment and patient care [1].

### 1.2. Etiological Factors of Breast Cancer

Two probable theories of initiation and progression of breast cancer are proposed: the cancer stem cell theory and the stochastic theory [11]. The first theory suggests that all subtypes of cancer originate from the same stem cells or progenitor cells due to acquired genetic mutations. The stochastic theory, on the other hand, suggests that different subtypes of cancer are initiated by a single type of cell due to random mutations. However, neither of these theories fully explains the origin and mechanism of breast cancer formation [12]. Therefore, breast cancer is considered a multifactorial disease. Major risk factors for breast cancer are presented in Table 1.

The most significant risk factors for developing breast cancer are age and gender. With age, the risk of breast cancer increases for both women and men. Around 80% of breast cancer cases occur in women over the age of 50, with the median age of diagnosis being 62 years [13]. According to a report by Siegel et al., in 2016 in the United States, approximately 99.3% of all breast cancer deaths occurred in women over the age of 60 and 71.2% of all breast cancer deaths occurred in women over the age of 40 [14]. According to data from the American Cancer Society, there has been a sharp increase in the incidence of breast cancer among white women under 50 (1.4% per year) compared to women aged 50 and older (0.7%) [15]. The probable cause of increased risk with age is the accumulation of genetic mutations, epigenetic changes and the progressive aging of cells, which increase susceptibility to carcinogenesis.

Breast cancer occurs more frequently in women. It is estimated that less than 1% of breast cancer patients are men, but it is important to note that, over the past thirty years, there has been a significant increase in cases among men [16,17].

Race seems to be an important risk factor for breast cancer. According to Ban et al., the incidence of this cancer in Caucasian women is approximately 127.4 per 100,000 people and 121.4 per 100,000 in Black women. It has also been shown that breast cancer incidence is significantly lower among Latinas, and the disease is diagnosed at a younger age [18]. Similarly, lower incidence rates have been recorded in Asians and Native Alaskans [13].

Meo et al. [19] in a large review study found that women with blood group A and a positive Rh factor have a higher risk of developing breast cancer, while women with blood group AB and a negative Rh factor are in a lower-risk group. Similar observations were made by Saxena et al. [20], but Gates et al. [21] did not observe a clear association between blood group serology and breast cancer risk as well as its subtypes [20,21]. The mechanism of the relationship between blood group and Rh factor and breast cancer frequency has not yet been established, though genetic factors are suggested to be involved.

Breast cancer is a disease with a multigenic etiology. Both mutations and abnormal amplification of oncogenes and tumor suppressor genes play a key role in the initiation and progression of the disease. According to recent data, 15–25% of breast cancers are inherited among individuals with relatives susceptible to cancer. *BRCA1*, *BRCA2*, *TP53, CDH1*, *PTEN* and *STK11* are the most common genes involved in familial breast cancer, which occurs in 80% of cases. In approximately 2–3% of cases, mutations in genes with moderate predisposition such as *CHEK2*, *BRIP1*, *ATM* and *PALB2* are present. On the other hand, mutations in genes predisposed to low-penetrance cancers include *FGFGR2*, *LSP1*, *MAP3K1*, *TGFβ1*, *TOX3* and *MSH6* [22].

The most well-known genes are those related to breast cancer 1 and 2 (*BRCA1* and *BRCA2*). These genes belong to the group of tumor suppressors and code for tumor-suppressive proteins that regulate cell growth [12]. Mutations in these genes are inherited in an autosomal dominant manner. The *BRCA1* gene is located on chromosome 17 (17q21) and encodes a nuclear phosphoprotein with a molecular weight of 220 kDa and 1863 amino acids in 24 exons [23]. On the other hand, these exons are divided into the N-terminal RING domain, a fingerprint of the protein and the C-terminal BRCT domain, each playing a key role. A deficiency in BRCA1 can lead to disruptions in the regulation of cell cycle checkpoints and abnormal centrosome duplication, which in turn can cause genetic instability and ultimately apoptosis [24]. Women with *BRCA1* mutations are at an increased cumulative risk of breast cancer, ranging from 44% to 78% [25]. The *BRCA2* gene is located on chromosome 13 (13q12). The BRCA2 protein regulates the repair of double-strand DNA breaks through interactions with RAD51 and DMC1 [26,27]. In the case of *BRCA2* mutation carriers, the cumulative risk of breast cancer is 31–56% [25]. Locations of the common genes involved in familial breast cancer are presented in Table 2.

The *HER2* gene (Human Epidermal Growth Factor Receptor 2) is located on the long arm of chromosome 17 (17q12). *HER2* expression is primarily activated through gene amplification and rearrangement. This gene encodes a cell surface receptor for epidermal growth factor, which controls cell growth and division. In breast cancer, overexpression of this gene is associated with more aggressive tumor growth, but it can also be a target for therapy [12].

The *TP53* gene is located on chromosome 17p13.1 and encodes the phosphoprotein p53. Mutations in p53 disrupt transcriptional processes, affecting DNA repair, cellular aging, apoptosis, autophagy and angiogenesis processes. The *CHEK2* gene is located on chromosome 22q12.1 and belongs to the tumor suppressor gene group. This gene plays a crucial role in DNA repair, cell cycle regulation and apoptosis [22]. Identification of pathogenic genetic variants suggests intensive monitoring of the patient and enables the implementation of appropriate preventive measures.

One of the risk factors for malignant breast cancer is also the exposure of breast glandular tissue to endogenous ovarian hormones—mainly estrogens and progesterone. Therefore, an early age of menarche (before 12 years of age) and a late menopause (after 55 years of age) may be associated with an increased risk of breast cancer. Estrogens influence the proliferation of mammary gland epithelial cells, leading to an increased risk of replication errors and mutations. Genomic instability and an increased risk of malignant transformation are also influenced by changes in inflammatory mediators and growth factors occurring under the influence of estrogens. Thus, the longer the exposure time, the greater the risk of developing breast cancer [25]. A systematic review of 117 epidemiological studies published in 2012 found that the risk of breast cancer increases by 5% for each year earlier menarche occurs (with the cutoff point set at 13 years of age) and by 2.9% for each year of delay in menopause after 51.5 years of age [28]. According to Fortner et al., who conducted the Nurses’ Health Study II, circulating estrogen levels are important for breast cancer after menopause; however, studies in large groups suggest that estrogens may also be linked to the occurrence of breast cancer in premenopausal women [29,30]. It has also been shown that exogenous estrogens found in oral contraceptives and hormone replacement therapy can increase the risk of breast cancer [25].

Another reproductive risk factor is having a child, as well as the age of the first pregnancy. Women who have never given birth are at higher risk of developing breast cancer than multiparous women. It has been shown that pregnancy before the age of 20 significantly reduces the risk of breast cancer, while the protective effect of pregnancy becomes insignificant after the age of 30 [25].

Certain benign changes occurring in the breasts may be associated with a 70% higher risk of developing breast cancer compared to women without any breast changes. Special attention should be given to changes involving atypical proliferative growth, such as atypical ductal hyperplasia (ADH) and atypical lobular hyperplasia (ALH). In an observational study by Hartmann et al., it was shown that atypical hyperplasias were associated with a relative risk (RR) of 4.24, while for women with non-proliferative changes, the RR was 1.27 [31]. Additionally, the risk depends on the age of diagnosis and is higher for women in whom atypical changes were diagnosed before the age of 45 compared to older patients.

Another significant independent risk factor for breast cancer is breast density, defined as the percentage of epithelial and connective tissue in the breast visible on a mammogram. A meta-analysis by McCormack et al. showed a significant increase in the risk of malignant breast cancer with increasing breast density (1.79 for density 5–24% vs. 4.64 for >75%) [32]. It is assumed that high mammographic density is associated with a higher susceptibility to cancer due to increased epithelial–stroma interactions and hormonal influences.

Breastfeeding may be a potential protective factor against breast cancer. It has been shown that the protective effect of lactation increases with the duration of breastfeeding [33]. In a meta-analysis of 75 studies conducted by Mao et al., it was shown that breastfeeding was associated with a reduced risk of all analyzed subtypes of breast cancer [34]. It was observed that the risk of breast cancer decreases by 4.3% for every 12 months of breastfeeding. Breastfeeding also reduces the risk of triple-negative breast cancer (20%) and the risk of cancer in BRCA1 mutation carriers (22–55%) [35].

In recent years, there has been significant scientific interest in the relationship between vitamin D and cancer diseases. Similar observations have been made regarding breast cancer. The active form of vitamin D at the cellular level influences cell proliferation and differentiation, has antioxidant properties and enhances apoptosis, while at the tissue level it may inhibit angiogenesis in tumor tissue and affect the tumor’s ability to invade and metastasize. Vitamin D acts on cells through the VDR (Vitamin D Receptor), which functions as a transcription factor [36,37]. Polymorphisms of this receptor are associated with the risk of developing breast cancer [38]. According to Garland et al., vitamin D levels above 50 ng/mL may be associated with a reduced risk of breast cancer [39].

Obesity is a significant modifiable risk factor for the development of malignant breast cancer. Obesity plays a particularly detrimental role in postmenopausal women. In a meta-analysis by Renehan et al., it was shown that, in postmenopausal women, every 5 kg/m^2^ increase in BMI increases the risk of cancer by 12% [40]. Fat tissue in obese individuals exhibits metabolic imbalance in the function of adipocytes and macrophages, which can lead to changes in hunger regulation, increased circulating estrogens from the aromatization process, lipid storage and chronic inflammation, all of which may contribute to carcinogenesis.

Alcohol consumption has been shown to be associated with an increased risk of cancer in breast tissue. In fact, even a small dose of alcohol increases the risk of breast cancer [41]. Therefore, there does not seem to exist a safe dose of alcohol. According to British data, around 2000 new cases are linked to alcohol consumption. The exact mechanism of how alcohol mediates carcinogenesis has not been described, but it is suggested that alcohol affects hormonal balance and the significant role of its toxic metabolites. It is important to note that the negative effects of alcohol increase with the dose, and a safe dose has not been established [42].

There is also a suggested potential link between breast cancer and air pollution. Andersen et al., in a study involving 15 cohort studies from European countries, indicate a correlation between breast cancer incidence in postmenopausal women and air pollution, particularly regarding nitrogen oxide compounds (NOx) [42].

Other risk factors for the development of breast cancer include dietary factors, stress, and reduced physical activity, which will be discussed in later sections of the paper.

Proper identification of risk factors and an understanding of the mechanisms through which they lead to disease can significantly contribute to breast cancer prevention by avoiding specific risk factors and promoting health-promoting behaviors. Additionally, understanding the pathogenesis of breast cancer contributes to targeted diagnostics and identifying individuals in risk groups helps with the early detection of the disease.

## 2. Nutritional Determinants of Breast Cancer Etiology

### 2.1. Fats, Proteins, Carbohydrates

The correlation between macronutrient intake—protein, fat and carbohydrates—and breast cancer (BC) risk is undoubtedly of a great interest in clinical practice; however, there is not a clear understanding of their contribution to BC rates. Importantly, not only does their percent of energy in a diet matter, but it also seems like their content and sources in diets may be just as important. Moreover, in some studies, the results may vary on other factors like ethnicity, age and demographics. Nevertheless, as macronutrient intake may be easily modified, and therefore influence disease risk, this research area is of great interest. In this paragraph, we will discuss that correlation and the results coming mostly from meta-analyses, systematic reviews and randomized clinical trials.

#### 2.1.1. Dietary Fat

Dietary fats are among the key components of nutrition that may modulate the risk of breast cancer. Current evidence suggests that the impact of dietary fat on carcinogenesis is not uniform and depends on both the type of fatty acids consumed and the individual’s metabolic context, including menopausal status, body weight and overall dietary patterns. Moreover, prospective studies and metanalysis show the association between breast cancer incidence and a high-fat diet [43,44]. However, the quality of dietary fat is as essential, as epidemiological studies and meta-analyses indicate that a high intake of saturated and trans fats may be positively associated with increased BC risk, whereas polyunsaturated fatty acids (PUFAs), especially omega-3 fatty acids, may exert a protective effect [45,46,47,48,49,50]. However, findings, mostly from RCTs, remain inconsistent, complicating the interpretation of causal relationships.

Saturated fatty acids (SFAs) and trans fatty acids (TFAs) have been considered as increasing BC risk in many studies. In a meta-analysis of Lodi et al., total fat, SFAs, monosaturated fatty acids (MUFAs), polyunsaturated fatty acids (PUFAs) and cholesterol intake did not correlate with BC risk in the general population or pre-menopausal women, regardless of the study type [45]. However, a high consumption of SFAs in post-menopausal women significantly increased BC risk in case-control studies by 12% (RR: 1.12; 95% CI: 1.03–1.21; *p* = 0.006). Similar results were observed in Xia et al.’s meta-analysis, where a higher dietary SFA intake was not associated with risk of BC among premenopausal women, in cohort studies or hospital-based studies. However, a positive association between higher dietary SFA intake and postmenopausal BC risk was observed in case-control but not in cohort studies [49]. Further, in Boyd et al.’s meta-analysis, SFA intake significantly increased BC risk (RR, 1.19; 95% CI: 1.06–1.35) [48]. Moreover, meat intake also significantly increased breast cancer risk (RR, 1.17; 95% CI 1.06–1.29) in this study. In a Smit-Warner et al. analysis of cohort studies, a very weak association was observed in increased BC incidence when saturated fats were substituted with carbohydrates (RR = 1.09 (CI = 1.00–1.19). Moreover, none of the other types of fat examined were significantly associated with breast cancer risk relative to an equivalent reduction in carbohydrate consumption [47]. Moreover, according to the Sieri et al. study, with 11.5 years of follow-up, the negative effect of SFA consumption may be observed in receptor-positive BC, as the authors observed that high SFA intake particularly increased the risk of this BC subtype [50]. A similar correlation was suggested in another study [51]. Further, SFA intake may not only increase BC risk, but it may also increase the risk of breast cancer-related death. According to the meta-analysis of Brennan et al., the highest intake of saturated fat increased risk of BC death when compared with the lowest SFAs intake (HR = 1.51; 95% CI: 1.09, 2.09) [46].

A similar negative effect on BC risk can be observed in TFAs, although the results are not that consistent. For example, in the Nurses’ Health Study II, fatty acids did not correlate with BC risk overall, but heterogeneity was observed by body mass index (BMI). Total SFA intake inversely correlated with BC risk among women with normal body weight, but among women with excessive body weight it significantly increased BC (OR = 2.33, 95% CI = 1.45–3.77, *p* = 0.007) [52]. In another meta-analysis, TFAs also did not correlate with BC risk; however, this study showed that the serum levels of trans fats could be associated with an increased risk of breast cancer only among postmenopausal women (pooled effect size: 1.37, 95% CI: 1.04–1.81, *p* = 0.02) [53].

When it comes to PUFAs, in the already mentioned NHS II study, PUFAs significantly decreased BC risk among women with excessive body weight (OR = 0.57, 95% CI: 0.36–0.89, *p* = 0.003) [52]. Moreover, a high intake (>73 mg/d) of eicosapentaenoic acid (EPA) and docosahexaenoic acid (DHA) from marine sources (not fish oil supplements) reduced the risk of additional breast cancer events approximately by 25% when compared with the lowest tertile of intake [54]. Similar results were observed in Zheng et al.’s meta-analysis, where an intake of marine n-3 PUFAs, but not alpha linolenic acid (ALA), was associated with a 14% lower risk of breast cancer [55]. Several studies suggested that an increased n3:n6 ratio may have a favorable effect on BC risk, suggesting a negative effect of several n-6 fatty acids [56]. However, other studies did not confirm this results [57]. In general, optimizing the n-6:n-3 ratio may have preventive potential, but current evidence is insufficient to support definitive population-wide recommendations.

#### 2.1.2. Dietary Protein

Evidence on the association between dietary protein intake and breast cancer (BC) risk remains inconclusive; however, an increasing number of studies suggest that the source of protein may be more relevant than total intake.

The Women’s Health Initiative study conducted by Pan et al. showed that a higher intake of plant protein was associated with a reduced risk of BC (HR = 0.98, 95% CI = 0.96–0.99, *p* = 0.006), while animal protein was associated with an increased breast cancer incidence (HR = 1.03, 95% CI = 1.01–1.06, *p* = 0.02) [58]. Neither animal nor plant protein affected breast cancer-related death in this study. Although these observations support the protective role of plant-based dietary patterns, not all studies provide the same results. For instance, an umbrella review by Kühn et al. reported no significant association between total protein intake (from either plant or animal sources) and breast cancer risk [59]. The authors emphasized the moderate quality of available evidence, which was limited by variability in dietary assessment, lack of intake standardization and heterogeneous populations.

In addition, a cohort study by Jahromi et al. demonstrated that high-protein dietary patterns, especially those low in either carbohydrates or fats, may have a protective effect. High-protein, low-carbohydrate diets were associated with a 29% lower risk of BC (OR = 0.71; 95% CI: 0.56–0.90, *p* = 0.005), and high-protein, low-fat diets with a 24% lower risk (OR = 0.76; 95% CI: 0.60–0.97, *p* = 0.026). Moreover, substituting fats with protein or replacing refined carbohydrates with plant protein may contribute to decreased risk of BC by 20 or 50% and 33% or 66%, respectively (depending on kilocalories replacement, 50 vs. 100 kcal, respectively).

In a meta-analysis by Wu et al., a high intake of red meat was significantly associated with an increased risk of breast cancer (RR = 1.07; 95% CI: 1.01–1.14) in a dose–response manner, particularly for fresh red meat (RR = 1.13; 95% CI: 1.01–1.26) and processed meat (RR = 1.09; 95% CI: 1.02–1.17). In contrast, soy consumption (RR = 0.91; 95% CI: 0.84–1.00) and skimmed milk (RR = 0.96; 95% CI: 0.92–1.00) were associated with a lower risk. Intake of poultry, fish, eggs, nuts, and total and whole milk were not correlated with BC risk.

Moreover, it should be remembered that a higher overall protein intake may also support improved outcomes in patients with BC. In the Nurses’ Health Study, a higher total protein intake modestly improved overall survival, regardless of the expression of insulin receptor status [60]. No specific protein-related nutrients, e.g., amino acids, were responsible for that observation in this study.

Nonetheless, long-term, well-controlled randomized trials comparing specific protein sources are still lacking and are necessary to clarify these associations, particularly when accounting for hormone receptor status, menopausal stage and overall dietary patterns.

#### 2.1.3. Dietary Carbohydrates

Current evidence on the association between carbohydrate intake, glycemic index, glycemic load and dietary fiber and breast cancer risk is inconsistent and does not allow for definitive conclusions.

In a meta-analysis by Fabiani et al., including 33 prospective studies with over 1.8 million participants and 13,336 breast cancer cases, a modest association between high dietary GI and increased BC risk in postmenopausal women (RR = 1.06; 95% CI: 1.00–1.12) was observed [61]. This association was slightly stronger in women with ER− and/or PR− tumors, although not statistically significant (RR = 1.11; 95% CI: 0.99–1.25). Moreover, no significant associations were found for glycemic load, total carbohydrate intake (112.3–343.5 g/day) or sugar/fructose consumption across menopausal groups. Notably, after stratifying by hormone receptor status, high GI diets were significantly associated with increased BC risk among women with hormone receptor-negative tumors, both pre- and postmenopausal. The association was not modified by BMI, suggesting an effect independent of adiposity.

On the other hand, a study by Holmes et al. did not confirm those results [62]. No significant associations were found between the intake of carbohydrates, total and specific fiber types, GI or GL and BC risk. The relative risks comparing the highest to the lowest quintiles were 0.97 for carbohydrates, 1.08 for GI, 0.99 for GL and 0.98 for fiber, with all confidence intervals crossing unity. Even among women with extreme fiber intakes (>30 g/day vs. ≤10 g/day), no statistically significant risk reduction was observed (RR = 0.68; 95% CI: 0.43–1.06). Stratified analyses by BMI and menopausal status also showed no clear risk patterns.

At present, there is insufficient justification to recommend reducing total carbohydrate intake solely for the prevention of breast cancer. However, in women—particularly postmenopausal women and those at risk for hormone receptor-negative tumors—it may be reasonable to consider limiting high glycemic index diets, for example, by reducing the intake of highly processed foods rich in refined starches and simple sugars.

### 2.2. Macroelements

Two of the most important macroelements for breast cancer risk, development and course seem to be calcium and magnesium.

#### 2.2.1. Calcium

Calcium is one of the macronutrients whose main sources are, among others, dairy products, sesame seeds or green leafy vegetables [63]. Observational studies suggest the relationship between calcium intake and breast cancer risk. The protective effects of calcium on breast carcinogenesis are probably caused by the antiproliferative properties of calcium. However, data about the impact of calcium intake on the risk of breast cancer is still unclear [64]. A study among the Chinese population showed a negative association between calcium intake and the risk of breast cancer. However, there are no associations between dairy products and the risk of this cancer [64]. Wu et al., in a pooled analysis of 21 cohort studies, reported that calcium and dairy product intake are not linked to a higher risk of breast cancer. On the other hand, higher intake of cottage and ricotta cheese and yoghurt is inversely associated with ER-negative breast cancer risk [65]. Interesting results were presented by Fernendez-Lazaro et al. They showed that the association between calcium intake and breast cancer is L-shaped, with the vertex of this curve around 1200–1400 mg calcium per day [66]. On the other hand, Hong et al. suggested the lowest risk of breast cancer is among women who intake 600 mg of calcium per day [67]. A dose–response meta-analysis showed that the risk of breast cancer was reduced by 1% in each 350 mg per day increment in dietary calcium and by 6% in each 350 mg per day increment in total calcium [68]. An Iranian study showed that dietary calcium intake has a protective effect in breast cancer, but only in premenopausal women (not in postmenopausal) [69]. It is also vital to note that dairy products, the main source of calcium, have an impact on breast cancer. However, this impact depends on the type of dairy product, subtype of breast cancer and menopausal status. Dairy products decrease risk of estrogen receptor-positive and progesterone receptor-positive cancer. Fermented dairy products are especially beneficial for postmenopausal women because their intake reduces the risk of breast cancer. On the other hand, premenopausal women should intake low-fat dairy, which reduces the risk of breast cancer in this population [70]. We need future studies focused on the association between various groups of dairy products (including fermented dairy products) and breast cancer.

#### 2.2.2. Magnesium

Another macronutrient that plays an important role in breast cancer development seems to be magnesium. Huang et al. showed that a higher magnesium intake is associated with a lower risk of breast cancer. The mediating role in the association between magnesium intake and breast cancer risk was played by CRP level [71]. Additionally, women with breast cancer present disrupt in magnesium homeostasis. The level of plasma, ionized and erythrocyte magnesium levels is lower in women suffering from breast cancer when compared to healthy women. Moreover, the women with cancer intake lower amounts of magnesium than healthy women [72]. Moreover, magnesium intake may improve survival in breast cancer [73].

### 2.3. Fruits and Vegetables, Vitamins

Vegetables and fruits are foods with a high nutritional density, and their consumption provides a variety of vitamins, including vitamins C and E, flavonoids—over five thousand polyphenolic compounds [74]—and fiber. At the same time, they are the basis of the so-called healthy dietary models, including the Mediterranean diet or the Asian diet, the consumption of which is associated with a beneficial effect on health [75]. Meta-analyses also show an inverse association between the vegetable–fruit-based dietary pattern and breast cancer risk [76,77,78,79,80,81].

In the meta-analysis of 15 studies, including 7071 cases of breast cancer, it was observed that each additional intake of 100 g of fruit (RR = 0.97, 95% CI, 0.95–0.99) and each additional intake of 100 g of vegetables (RR = 0.97, 95% CI, 0.95–0.99), was associated with a small decrease in breast cancer risk [76].

Of note is the systematic review and meta-analysis of prospective studies by Farvid et al. from 2021 [82], which found that total fruit and vegetable intake is associated with a lower risk of total breast cancer and postmenopausal breast cancer, as well as estrogen- and progesterone-receptor-positive (ER+/PR+) and negative (ER-/PR-) breast cancer. Yellow/orange and cruciferous vegetables were not significantly associated with breast cancer risk. In addition, there was no significant association between green leafy vegetables and tomatoes and breast cancer risk. In turn, it has been shown that a high intake of fruit juices can increase the risk of breast cancer [82].

Quite surprisingly, the negative impact of fruit juices is probably caused by the high content of simple sugars, the consumption of which promotes the development of excessive body mass and carbohydrate metabolism disorders, which may indirectly increase the production of pro-inflammatory cytokines. At the same time, despite the content of antioxidants and vitamins in them, juices are poor in dietary fiber, so it is recommended to eat the whole fruit instead of the juice [82].

Dietary fiber is a desirable part of every woman’s diet. It positively impacts gut microbiota and can reduce the activity of intestinal β-glucuronidase, which is necessary for the hydrolysis of conjugated estrogens, resulting in less estrogen reabsorption and higher amounts excreted from the body [83].

In addition, a higher intake of fiber promotes the prolongation of the transit of food through the small intestine, as a result slowing down the increase in postprandial glycemia and hyperinsulinemia. High-fiber diets are also associated with a lower risk of overweight and obesity, due to their satiating properties [84,85].

In a meta-analysis of prospective studies, Aune et al. showed an inverse association between dietary fiber intake, especially soluble fiber, and breast cancer risk; however, this association seemed to be most pronounced in studies with high levels (≥25 vs. <25 g/day) or large ranges (≥13 vs. <13 g/day) of fiber intake [84].

In addition to fiber, vegetables and fruits are characterized by an extremely high content of flavonoid compounds with antioxidant and anti-inflammatory properties, of which flavonols—e.g., quercetin, kaempferol and myricetin present in onions, broccoli, tea and fruit—and flavones—e.g., luteolin, apigenin and tangeretin present in fresh herbs, celery or chamomile tea—are associated with a lower risk of breast cancer, especially among postmenopausal women [74].

Moreover, the results of a 2022 meta-analysis by Liu et al. suggest that the intake of flavonols at around 14.5~60 mg/d and flavones at more than 0.2~31 mg/d may have a preventive effect on breast cancer [86].

The mechanism of action of flavonoids results from their strong antioxidant activity, which directly interferes with the initiation, promotion and development of cancer, and indirectly interferes due to their effect on hormonal activity, e.g., aromatase that converts androgens into estrogen. Additionally, some phytoestrogens, such as genistein, apigenin, naringenin and kaempferol, can bind to estrogen receptors and compete with 17β-estradiol in binding to them. Moreover, daidzein, genistein, quercetin and luteolin have the ability to inhibit proliferative activity induced by environmental estrogen, which suggests antiestrogenic and anticancer functions of individual flavonoid compounds [86].

The issue of soy consumption, which is a rich source of soy isoflavones including daidzein and genistein, is also very interesting. Kazemi et al., based on data from seven studies with 4055 cases of breast cancer, observed that each additional consumption of soy and/or soy products by 30 g/d was associated with a 3.5% reduction in breast cancer risk (RR = 0.965; 95% CI, 0.94–0.9). However, no significant associations were observed with the consumption of other legumes [76].

The relationship between the consumption of individual components of vegetables and fruits and the risk of breast cancer was also studied. Vitamin C, ascorbic acid, is a water-soluble vitamin with extremely strong antioxidant properties. Interestingly, reports on the link between vitamin C and cancer are contradictory [81,87,88,89,90].

A 2024 meta-analysis found an inverse association between dietary vitamin C intake and breast cancer risk in case-control studies, but not in cohort studies. There was also no association between vitamin C supplementation and breast cancer risk [91].

In contrast, a meta-analysis that included 10 studies, with 17,696 cases of breast cancer, suggested that dietary and supplement intake of vitamin C may be associated with a reduced risk of breast cancer mortality; supplementation was associated with a 15% lower risk of death from breast cancer, and dietary vitamin C intake—for every 100 mg/day more—was associated with a 22% lower risk of death [92].

Some researchers indicate that dietary vitamin C intake may be more beneficial due to other components and compounds co-occurring in food sources that may potentially contribute synergistically to cancer prevention [91,93].

The second strong antioxidant that is associated with anti-cancer and chemopreventive activity is vitamin E [94].

Despite this, the available meta-analyses do not indicate an association between vitamin E intake and a reduced risk of breast cancer [88,95].

However, a 2023 meta-analysis showed that vitamin E intake was inversely associated with breast cancer recurrence, although no association was found with breast cancer mortality [95]. Furthermore, in a meta-analysis by Hu et al., it was suggested that severe α-tocopherol deficiency may increase the risk of breast cancer [89].

Vegetables and fruits, especially in their raw form, are also a great source of folic acid and other B vitamins. A meta-analysis of 27 studies, including 49,707 breast cancer cases, indicated that a high intake of folic acid, vitamin B6 and vitamin B2 may reduce the risk of breast cancer. No significant association was found for B12. Further studies have shown that folate and vitamin B6 may reduce the risk of ER-/PR- breast cancer, but not ER+/PR+ [96].

Similarly, a meta-analysis of 39 studies on folate intake and 12 studies on plasma folate levels found that folate intake was inversely correlated with breast cancer risk, especially in premenopausal women, but plasma folate levels were not significantly associated with risk [97].

A meta-analysis of 23 prospective studies found that folate may reduce the risk of ER- and ER-/PR- breast cancer, and that its effects may be particularly beneficial among premenopausal women and women with moderate to high alcohol consumption [98].

Finally, vitamin D, with its pleiotropic action, cannot be overlooked. The results of a meta-analysis (2019) of 22 observational studies showed that total vitamin D intake and vitamin D supplementation are inversely associated with the occurrence of breast cancer, and its deficiency is directly related to the occurrence of breast cancer [99]. In contrast, another meta-analysis with a systematic review suggested that a protective association between blood 25(OH)D levels and the development of breast cancer occurs in premenopausal but not postmenopausal women [100].

It appears that women diagnosed with breast cancer also have lower vitamin D concentrations than women in the control group [101,102].

Contrary to the mentioned studies, Zhou et al. evaluated whether vitamin D supplementation, with or without calcium, reduced the risk of breast cancer by at least 30%, which was taken as the threshold for clinically significant benefit. The assumed association was not observed, suggesting that vitamin D supplementation does not significantly reduce the risk of breast cancer [103].

Interestingly, however, there are suggestions that pre-treatment vitamin D deficiency is associated with a worse response to neoadjuvant chemotherapy in breast cancer patients, although further prospective studies in this area are recommended [104].

The latest epigenomic, transcriptomic and proteomic studies provide more and more information about the mechanisms by which vitamin D affects the regulation of self-renewal, differentiation, proliferation, transformation and death in cancer cells. The relationship between the immune system and the anti-cancer properties of vitamin D is also indicated [105].

The summary of nutritional factors that decreased the risk of breast cancer is presented in Table 3, while factors that linked to survival in breast cancer are shown in Table 4.

### 2.4. Nutritional Guidelines in Breast Cancer

Proper nutrition in cancer is an essential element of treatment. According to estimates, about 10–20% of patients with cancer die due to malnutrition. The European Society for Clinical Nutrition and Metabolism recommends supplying energy between 25–30 kcal/kg/day, with protein intake above 1 g/kg/day. Oral intake, including oral nutritional supplements, is preferred for patients who can eat but are malnourished or at risk of malnutrition [106].

It is vital to notice that n-3 fatty acids, especially docosahexaenoic acid and eicosapentaenoic acid, improve the nutritional status and course of disease in women with breast cancer [107,108]. Moreover, docosahexaenoic acid may improve the chemotherapy outcome [109]. For this reason, patients with breast cancer should care about n-3 fatty acid intake with food, including oral nutritional supplements enriched in n-3 fatty acids, or introduce proper supplementation.

Agent, which is often used in the therapy of women with hormone receptor-positive early breast cancer, is an aromatase inhibitor, which may cause bone loss. Prieto-Alhambra et al. presented that improving the vitamin D status may inhibit the aromatase inhibitor-related bone loss. Additionally, the authors observe that the concentration of 25OHD of 20 ng/mL is too low to maintain bone health [110]. In fact, about 66% of women with breast cancer have vitamin D deficiency or insufficiency, which is associated with a reduced bone mineral density of the lumbar spine [111]. Therefore, it seems appropriate to assess the vitamin D status in all patients with breast cancer, and to perform personal dose titration.

A diet that is recommended for breast cancer prevention is the Mediterranean diet. Among patients with a diagnosis of breast cancer, the Mediterranean diet may also be beneficial. Firstly, this diet decreases the risk of tumor recurrence after treatment. Secondly, it may improve the patients’ quality of life. The Mediterranean diet presents anti-inflammatory, antioxidant, anti-proliferation and pro-apoptotic effects. It also induces autophagy and positively affects gut microbiota and lipid profile [112]. However, some patients may have some limitations in introducing the Mediterranean diet. This diet contains many products such as fresh vegetables and fruits, grains or wholegrain bread [113], which patients poorly tolerate, especially during therapy. Therefore, the Mediterranean diet may need to be personalized.

Therapy of breast cancer often causes side effects, such as vomiting, loss of appetite, nausea or changes in taste, which lead to a decrease in intake [114]. Therefore, diet should provide all nutrients and reduce the symptoms related to treatment. Zinc supplementation may benefit patients with chemotherapy-related taste disorders. Additionally, chewing gum or lemon juice intake before a meal may also help with this problem [115]. Patients with gastrointestinal symptoms should choose creams with unrefined rice, some cooked vegetables and miso soup [114].

## 3. Microbiota and Cancer

### 3.1. Microbiota Dysbiosis and Predisposition to Breast Cancer

The established and well-known risk factors for breast cancer in many women do not fully explain the development of this malignancy in a large group of patients. The influence of genetic and environmental factors does not entirely elucidate the onset of this disease in genetically identical individuals subjected to the same environmental conditions. Furthermore, alterations in the gut microbiota may affect not only disease progression, but also its treatment and potential recurrence. A partial explanation is connected with the random occurrence of mutations, DNA methylation, lifestyle-related modifications, the presence of obesity and overweight, as well as physical activity, all of which directly influence the gut microbiome. Therefore, there is an ongoing search for additional markers of breast cancer risk. The pathogenic relationship between the composition of the microbiota and breast cancer typically involves taxonomic changes occurring within the gut; however, this relationship is also supported by alterations observed in breast tumor tissue compared to healthy individuals [116]. However, it is essential to evaluate the mechanisms through which the gut microbiota may influence the development of this cancer.

The primary mechanism in the pathogenesis of breast cancer involves the impact of microbiota on estrogen metabolism, as well as the development of a micro-inflammatory state, changes in gene expression and modifications of the immune system. The liver is the main organ involved in estrogen metabolism, where estrogens are conjugated and excreted into the intestinal lumen along with bile. The deconjugation of estrogens occurring in the intestines under the influence of bacterial β-glucuronidases is dependent on the diversity and taxonomy of gut bacteria. Subsequently, there is a reabsorption of free estrogens and their transport in a modified form to target tissues such as the mammary gland [117]. The relationship between the abundance of different bacterial strains and the concentration of β-glucosidase may significantly influence estrogen metabolism in the body [118]. In addition, other estrogen-like metabolites, xenoestrogens, as a result of the action of beta-specific b-glucuronidases, may remain in the body for a longer period of time, which significantly disrupts the body’s estrogen metabolism [119]. In postmenopausal women with breast cancer, however, strain diversity was independent of estrogen levels. They showed increased numbers of Clostridiaceae, Faecalibacterium and Ruminococcaceae strains, along with reduced numbers of Dorea and Lachnospiraceae [120].

The composition of the intestinal microbiota is significantly different in people diagnosed with breast cancer. This may also be influenced by long-term antibiotic therapy and chronic treatment used in this cancer. Studies involving women with breast cancer have shown that long-term antibiotic therapy reduced the diversity of their microbiota [121]. In the study by Wang et al., a decrease in Methylobacterium and an increase in Corynebacterium, Staphylococcus, Actinomyces and Propionibacteriaceae were observed in patients with invasive breast cancer compared to healthy controls [122]. Women diagnosed with breast cancer also had significantly lower levels of certain bacteria, such as Lactococcus and Streptococcus, which have anti-cancer properties [123]. Scientific studies have observed not only changes in the intestinal microbiota between healthy individuals and those with breast cancer, but also between different stages of disease advancement [124,125].

In overweight and obese women with breast cancer, Firmicutes, Faecalibacterium prausnitzii and Blautia spp. were reduced compared with normal-weight patients. In contrast, patients with stage 2/3 tumors had an increase in the total number of Bacteroidetes compared with patients with stage 0/1 tumors [124]. Differences between women with breast cancer and benign breast lesions and women without breast lesions were demonstrated not only in increased diversity but also in the occurrence of individual species. Escherichia and Lactobacillus were more common in women with benign breast lesions [126,127]. In addition, the intestinal metagenomes of postmenopausal breast cancer patients are rich in genes encoding the biosynthesis of lipopolysaccharide, which is a potent trigger of systemic inflammation, which may promote the process of neoplastic transformation [128]. A gradual increase in Bacteroidetes levels was observed with tumor progression, and low levels of Lachnospiriceae and Ruminococcus correlated with an increased risk of cancer recurrence [129]. The status of the microbiota also depends on other factors such as BMI, menopausal status or physical activity. However, the data on this subject are ambiguous. In women, a significant reduction in microbiota diversity was observed in the cancer group. In the BMI group, no statistically significant difference was observed between overweight and obesity [130].

The intestinal microbiota also plays an important immunomodulatory role, through interaction with antigen-presenting cells (dendritic cells) and interactions with Toll-like receptors (TLRs). Through the interaction with bacterial lipopolysaccharide (LPS) on the outer membrane of Gram-negative bacteria, it activates Toll-like receptor 4 (TLR4) on the host cell surface, inducing an immune response of T cells against cancer cells [131].

Systemic interactions between the microbiota and components of the immune system (IL-6 and neutrophils) and their role in the development of breast cancer are also known [132,133]. The microbiome, by maintaining the integrity of the gut wall, reduces the level of pro-inflammatory cytokines in epithelial cells of tissues such as the mammary gland [119]. A healthy gastrointestinal microbiome promotes the maturation of CD8 + NK T cells that can eliminate HER2/neu + breast tumor cells [134].

### 3.2. Microbiota Modulation and Probiotic Therapy During Cancer Treatment

The prevalence of differences in the gut microbiota of women with breast cancer raises the question of whether modifications in the microbiota through prebiotics and probiotics could influence disease progression and treatment processes. A primary dietary component affecting the composition of the microbiota is dietary fiber. By modulating the activity of β-glucuronidase in postmenopausal women with breast cancer, dietary fiber may affect estradiol levels [135]. SCFAs (especially butyrate) produced by the bacterial fermentation of dietary fiber show anticancer activity in animal models [136]. Combination therapy with sodium butyrate, tamoxiferon and 5-aza-2′-deoxycytidine (5-aza-CdR) was most effective in tumor suppression and in inducing the apoptosis of breast cancer cells [137]. Epigallocatechin-3-gallate (EGCG) (a polyphenol found in tea) treatment increased the efficacy of radiotherapy in breast cancer patients [138]. The use of probiotics in women with breast cancer may also have a systemic effect by reducing anthropometric parameters (BMI) and waist circumference (WC). However, inflammatory parameters such as TNF-α and hs-CRP concentrations did not decrease after the intervention [139]. Probiotic supplementation in rats significantly improved cognitive functions impaired in association with chemotherapy for breast cancer by changing the composition of the microbiota and modulating its metabolites. Increased levels of the metabolites p-Mentha-1,8-dien-7-ol, linoleaidylcarnitine and 1-aminocyclopropane-1-carboxylic acid negatively correlated with the occurrence of chemotherapy-related cognitive impairment [140]. Other studies have shown that probiotics inhibit tumor growth and reduce their size, which is attributed to their immunomodulatory and antiangiogenic properties. They have also been shown to reduce the number of metastases [141]. The gut microbiota plays a significant role in many metabolic processes, messenger pathways and the formation of modified metabolites. Its influence on the development of breast cancer and possible modifications to the course of this disease requires much scientific research.

## 4. Physical Activity and Breast Cancer

Research findings linking physical activity with malignant breast tumors are inconsistent. Most studies suggest a potential protective effect of regular physical activity, which may reduce the risk of developing breast cancer [142]. The observed inconsistency may stem from differing, unsystematic methods of assessing physical activity, variations in the type of activity and differences in the age of study participants. Currently, the most frequently studied form of exercise is aerobic training, though the role of resistance and combined training is also emphasized.

A meta-analysis by Monninkhof et al., including 19 cohort studies and 29 case-control studies, demonstrated an association between physical activity and postmenopausal breast cancer, with risk reductions ranging from 20% to 80%. Additionally, a 6% decrease in breast cancer risk was observed for each additional hour of weekly physical activity, assuming a consistent level of exercise [143].

In patients undergoing and following breast cancer treatment, exercise contributes to a reduction in mastectomy-related side effects by improving lymphatic drainage from the upper limbs. According to Schlienger et al., moderate-intensity aerobic exercise combined with resistance training provides benefits in early adjuvant breast cancer treatment by improving muscle strength and cardiopulmonary function [144]. Additionally, in patients with breast cancer, physical activity may contribute to reducing the risk of recurrence and lead to a decrease in overall mortality.

Chemotherapy-induced peripheral neuropathy is the most common neurological complication of chemotherapy. A meta-analysis by Duregon et al. showed that exercise significantly improved the quality of life in affected patients. Additionally, the greatest benefits were observed with programs combining endurance, strength and sensorimotor training [145].

The association between physical activity and quality of life in cancer patients has also been emphasized. A meta-analysis by Aune et al., involving 79 randomized controlled trials, demonstrated the positive impact of physical exercise on health-related quality of life (HRQoL) in breast cancer survivors. However, this effect was less pronounced regarding mental and emotional health [146]. 

Several mechanisms have been proposed to explain the relationship between physical activity and breast cancer. These include reductions in adipose tissue, lower exposure to insulin and insulin-like growth factors, increased intestinal motility, decreased estrogen exposure, reduced free radicals and a potential enhancement of immune defense against cancer [147]. Aerobic exercise has also been shown to influence angiogenesis by inhibiting pro-angiogenic marker expression. Additionally, physical activity may affect gene methylation and telomere lengthening, thereby reducing carcinogenesis risk [148]. In premenopausal women, physical activity may delay menarche and reduce the frequency of anovulatory cycles, contributing to lower circulating estrogen levels [25].

On the other hand, high-intensity and prolonged exercise is not recommended, as it may increase pro-inflammatory cytokine levels, leading to chronic inflammation, which in turn may raise the risk of cancer progression [149].

Further research is needed to determine the optimal type, frequency, duration and intensity of physical activity necessary to reduce the risk of breast cancer, improve the quality of life in patients with a breast cancer diagnosis and treatment history and decrease the risk of disease progression.

## 5. Stress and Breast Cancer

Psychological stress, especially chronic stress, significantly disrupts the body’s homeostasis, negatively affecting the functioning of the hormonal, nervous and immune systems. The main mechanism involves activation of the hypothalamic–pituitary–adrenal (HPA) axis, which leads to elevated levels of cortisol, catecholamines, chronic inflammation and immunosuppression. These phenomena may promote carcinogenesis, including the malignant transformation of breast cells [150].

Epidemiological studies indicate that stressful life events and chronic emotional stress may increase the risk of developing breast cancer. In a study by Chiriac et al. (2021), which included women with a history of severe stressors such as the loss of a loved one, violence or divorce, it was shown that they were 60% more likely to develop breast cancer than women without such exposure (OR = 1.6; 95% CI: 1.2–2.1) [151].

Stress not only affects the initiation of carcinogenesis but also its progression and prognosis. Activation of the sympatho–adrenal axis and an increase in proinflammatory cytokines (e.g., IL-6, TNF-α) result in impaired natural killer (NK) cell function, as well as increased angiogenesis, invasiveness and metastatic potential of cancer cells. In a systematic review by Gosain et al. (2020), it was noted that negative stress coping strategies and lack of social support are associated with higher mortality and shorter survival time [152].

Meta-analytic data indicate that patients with low social support and high stress levels face a 30–50% increased risk of death from breast cancer [151].

Another mechanism linking psychological stress to cancer progression is oxidative stress. The production of reactive oxygen species (ROS) leads to DNA, protein and lipid damage. In the study by Lee et al. (2017), it was demonstrated that oxidative stress biomarkers such as 8-OHdG and MDA are significantly elevated in patients with advanced breast cancer, and correlate with poorer prognosis and increased risk of recurrence [153].

A growing body of evidence shows that implementing effective stress-reduction strategies can not only enhance patients’ psychological well-being, but also improve clinical outcomes. Among effective interventions are cognitive–behavioral therapy, mindfulness meditation, physical activity and social support. In the study by Gosain et al., stress reduction programs based on these methods resulted in a significant decrease in cortisol levels and improved immune response (increased NK cell activity). For patients, this translates into better treatment outcomes and fewer complications associated with targeted cancer therapy [152].

## 6. Conclusions

There are many factors increasing risk of breast cancer, including genetic factors, especially *BRCA1*, *BRCA2*, *TP53*, *CDH1*, *PTEN* and *STK11* genes, and environmental factors, such as unbalanced diet, stress, low physical activity, air pollution or psychological stress. It is also vital to notice that gut microbiota may also influence breast cancer risk. Nevertheless, modification of some unhealthy habits may support therapy in breast cancer.

However, we need further studies regarding the impact of lifestyle elements on improving the prognosis of breast cancer patients, depending on the type of cancer or treatment.

Well-balanced diet, physical activity and strategies of coping with stress may be beneficial for patients and improve the efficacy of treatment as well as patients’ quality of life.

The most important guidelines relating to lifestyle, which support patients with breast cancer, are as follows:

1. Avoid alcohol.
2. Take care about your nutritional status—neither obesity nor underweight and malnutrition are recommended.
3. Well-balanced diet may improve your nutritional status: Change the sources of saturated and trans fatty acids to mono- and polyunsaturated fatty acids. Additionally, introduce products rich in protein, especially plant protein, and avoid meals with a high glycemic index.
4. Control your vitamin D status.
5. Try to eat vegetables and fruit, which are rich in polyphenols, but also are a good source of fiber—this is important for microbiota status.
6. Introduce physical activity according to your tolerance.
7. Find the relaxation techniques that are right for you to reduce emotional stress.

## Figures and Tables

**Table 1:** Major risk factors of breast cancer.

Risk Factors of Breast Cancer
Age
Gender
Genetic factors
Hormonal factors
Reproductive factors
Lifestyle (including diet, physical activity and stress)
Other cancer in the past
Benign changes
Breast tissue density
Ionizing radiation

**Table 2:** Location of the most common genes involved in familial breast cancer.

Gene	Location
*BRCA1*	17q21
*BRCA2*	13q12
*HER2*	17q12
*TP53*	17p13.1
*CHEK2*	22q12.1
*CDH1*	16q22.1
*PTEN*	10q23
*STK11*	19p13.3

*BRCA1*—Breast Cancer 1; *BRCA2*—Breast Cancer 2, *HER2*—Human Epidermal Growth Factor Receptor 2; *TP53*—Tumor Protein p53; *CHEK2*—Checkpoint Kinase 2; *CDH1*—Cadherin-1; *PTEN*—Phosphatase and tensin homolog; *STK11*—Serine/threonine kinase 11.

**Table 3:** The summary of nutritional factors that decreased the risk of breast cancer.

Factors That Decreased the Risk of Breast Cancer
Calcium
Magnesium
A diet rich in fruits and vegetables
Fiber
Vitamin C
Vitamin E
Vitamins B
Vitamin D

**Table 4:** The summary of factors associated with survival in breast cancer.

Factors Associated with Increased Survival in Breast Cancer	Factors Associated with Decreased Survival in Breast Cancer
Higher total protein intake	Saturated fatty acids intake
Magnesium intake	Negative stress coping strategies
Vitamin C intake	Lack of social support
Country (income country)	

## Data Availability

Not applicable.
